# Trauma treatment for children and adolescents: stabilizing or trauma-focused therapy?

**DOI:** 10.3402/ejpt.v6.27630

**Published:** 2015-04-08

**Authors:** Ramón J. L. Lindauer

**Affiliations:** 1 Department of Child and Adolescent Psychiatry, Academic Medical Centre, University of Amsterdam, Amsterdam, The Netherlands; 2 De Bascule, Academic Centre for Child and Adolescent Psychiatry, Amsterdam, The Netherlands

Child abuse is widely prevalent in the Netherlands, and the strong social and political focus now put on the issue is fully warranted. Prevalence studies have estimated that around 3% of all Dutch children experience some form of abuse or neglect (Alink et al., 2011; Lamers-Winkelman, Slot, Bijl, & Vijlbrief, 2007; Van IJzendoorn et al., 2007). The most common forms of maltreatment are neglect and domestic violence. Being a witness to domestic violence is a form of child maltreatment. In 2012, approximately 6,000 children under age 18 were accommodated in some kind of women's or homeless facility in the Netherlands. Half of these were in women's refuges after fleeing domestic violence. Research by Brilleslijper-Kater et al. (2010) found that such children had experienced an average of seven potentially distressing or traumatic events; most had directly experienced domestic violence by witnessing verbal or physical violence between their parents. A study in the Therapeutic Foster Care treatment program at De Bascule found that nearly a quarter of such children had experienced five or more different placements outside the parental home. Persistent relocations like these have an adverse impact on child development.

Maltreatment leads to serious problems in children's psychosocial functioning. Mental health problems may include posttraumatic stress disorder, other anxiety disorders, depressive disorder, attachment disorder, and conduct disorder. Such conditions are detrimental to a child's development and may culminate in problems in the family, problems at school, and problems with other children (Jonkman, Verlinden, Bolle, Boer, & Lindauer, 2013; Lindauer & Boer, 2012). The negative consequences may even persist into adulthood (Edwards, Holden, Anda, & Felitti, 2003); adults who have experienced abuse as children have higher risks of physical and psychological problems. Many mothers that experience intimate partner violence develop mental health problems like posttraumatic stress disorder, depressive disorder, or emotional regulation problems. Those problems, in turn, may affect their parenting competence and their children's psychosocial development. Such mothers are also more at risk of maltreating their own children (Dubowitz & Bennett, 2007). In sum, then, the prevalence of child abuse is high, and the short- and long-term consequences are severe.

The first objective of treatment is to create safety within the family. An end must be put to the traumatizing situation. That requires a multidisciplinary individual and systemic strategy that focuses both on the victim or victims and the perpetrator.

In forms of maltreatment such as violence or sexual abuse within the family, treatment must focus both on ensuring safety in the family and on assessing the childrearing capabilities of the parent or parents. Some questions that need to be asked are: (1) Do the parents acknowledge that their child has problems? (2) Do they acknowledge the part that they themselves have played in the onset of the problems? (3) Are they motivated in any way to try to change the situation? (4) Are the parents capable of recognizing experts as people that can truly help? (5) Do the parents themselves have mental health problems that complicate the treatment? Some situations are simply too unsafe, and some parents are utterly incapable of bringing up their child or children. That necessitates the placement of children away from home.

Once a safe environment has been created, the next question is whether the traumatized child should receive trauma-focused treatment right away or whether the first priority should be to ensure a degree of stability. There is considerable debate about this in the adult literature (e.g., De Jongh & ten Broeke, 2014; Dorrepaal et al., 2014). Several stabilization interventions for traumatized children and adolescents are available in the Netherlands, including the insight-therapeutic Stapstenen (“stepping stones”), the drama-therapeutic Tijd voor Toontje, the dissociation-focused Slapende Honden? Wakker Maken! (“don't let sleeping dogs lie”), and the Attachment, Self-Regulation and Competency (ARC) model, but their effectiveness has not been demonstrated. Some empirically validated trauma treatment modalities are trauma-focused cognitive-behavioral therapy (TF-CBT) and eye movement desensitization and reprocessing (EMDR; Diehle, Opmeer, Mannarino, Boer, & Lindauer, 2015); TF-CBT has been more thoroughly evaluated in randomized controlled studies. If a traumatized child or adolescent is broadly capable of talking about what he or she has experienced, then it is preferable to begin an evidence-based trauma intervention immediately without prior stabilization.
